# Research trends and hotspots on connectomes from 2005 to 2021: A bibliometric and latent Dirichlet allocation application study

**DOI:** 10.3389/fnins.2022.1046562

**Published:** 2022-12-22

**Authors:** Yangye Yan, Guoxin Fan, Xiang Liao, Xudong Zhao

**Affiliations:** ^1^Tongji University School of Medicine, Shanghai Eastern Hospital Affiliated to Tongji University, Shanghai, China; ^2^Department of Pain Medicine, Huazhong University of Science and Technology Union Shenzhen Hospital, Shenzhen, China; ^3^School of Biomedical Engineering, School of Medicine, Guangdong Key Laboratory for Biomedical Measurements and Ultrasound Imaging, Shenzhen University, Shenzhen, China; ^4^Department of Spine Surgery, Third Affiliated Hospital, Sun Yat-sen University, Guangzhou, China; ^5^Clinical Research Center for Mental Disorders, Chinese-German Institute of Mental Health, Shanghai Pudong New Area Mental Health Center, School of Medicine, Tongji University, Shanghai, China

**Keywords:** connectome, bibliometric, latent Dirichlet allocation, Web of Science, neuroscience

## Abstract

**Background:**

This study aimed to conduct a bibliometric analysis of publications on connectomes and illustrate its trends and hotspots using a machine-learning-based text mining algorithm.

**Methods:**

Documents were retrieved from the Web of Science Core Collection (WoSCC) and Scopus databases and analyzed in Rstudio 1.3.1. Through quantitative and qualitative methods, the most productive and impactful academic journals in the field of connectomes were compared in terms of the total number of publications and h-index over time. Meanwhile, the countries/regions and institutions involved in connectome research were compared, as well as their scientific collaboration. The study analyzed topics and research trends by R package “bibliometrix.” The major topics of connectomes were classified by Latent Dirichlet allocation (LDA).

**Results:**

A total of 14,140 publications were included in the study. NEUROIMAGE ranked first in terms of publication volume (1,427 articles) and impact factor (h-index:122) among all the relevant journals. The majority of articles were published by developed countries, with the United States having the most. Harvard Medical School and the University of Pennsylvania were the two most productive institutions. Neuroimaging analysis technology and brain functions and diseases were the two major topics of connectome research. The application of machine learning, deep learning, and graph theory analysis in connectome research has become the current trend, while an increasing number of studies were concentrating on dynamic functional connectivity. Meanwhile, researchers have begun investigating alcohol use disorders and migraine in terms of brain connectivity in the past 2 years.

**Conclusion:**

This study illustrates a comprehensive overview of connectome research and provides researchers with critical information for understanding the recent trends and hotspots of connectomes.

## 1 Background

“Connectome” is a subject that studies the operating mechanism of brain functions *via* analyzing the connection and organization patterns of neurons. With the development of brain imaging technology and computational science, such as the mapping of the human brain atlas, the advancement of Magnetic Resonance Imaging (MRI) data analysis techniques, and the integration with complex network theory, more possibilities for exploring human brain connectivity have been revealed (Sporns and Betzel, 2016). Scientific research in this field provided a holistic and multidimensional approach to understanding the brain’s operating mechanism and neuropsychological disorders (Sporns et al., 2005; Sporns, 2013). At present, research on the connectome is mainly focused on the large-scale level while constructing structural brain networks with structural MRI or diffusion MRI, or establishing functional brain networks with technologies such as functional MRI (fMRI), electroencephalogram (EEG), or magnetoencephalography (MEG) (Sadaghiani and Wirsich, 2020). Additional computer science and mathematics theories, such as machine learning and deep learning, were applied to connectome research. By combining complex network analysis methods based on graph theory and exploring its topological principles, we can further reveal the internal connection patterns and operating mechanisms of the human brain (Sporns, 2018; Farahani et al., 2019). The connectome provides a theoretical basis for brain development and neuropathology of neuropsychological diseases (van den Heuvel and Sporns, 2019). Therefore, summarizing the trends and hotspots of the connectome research may be instructive for further studies in the field.

Bibliometric analysis has become an ideal method for examining the literature in a certain scientific field over time. It can quantitatively analyze articles through mathematical and statistical methods (Guler et al., 2016). Through quantitative and qualitative analysis of the literature, it assists researchers in identifying the research trends and focus of a particular topic (Yan et al., 2020). Latent Dirichlet allocation (LDA) is an unsupervised machine-learning-based algorithm, which contains a three-layer probabilistic structure of words, topics and documents. LDA has been widely utilized as a bibliometric tool for identifying research topics and generating clusters of topic terms (Tran et al., 2019; Zhang et al., 2021).

There have been some bibliometric studies on brain imaging (Gong et al., 2019; Yan et al., 2020; Devezas, 2021), while few, to our knowledge, conducted the bibliometric analysis of connectomes. Moreover, most of the reviews on connectomes generally lack quantitative analysis. Therefore, it is necessary to carry out a bibliometric study on the connectome.

## 2 Materials and methods

### 2.1 Data acquisition and search strategy

**FIGURE 1:**
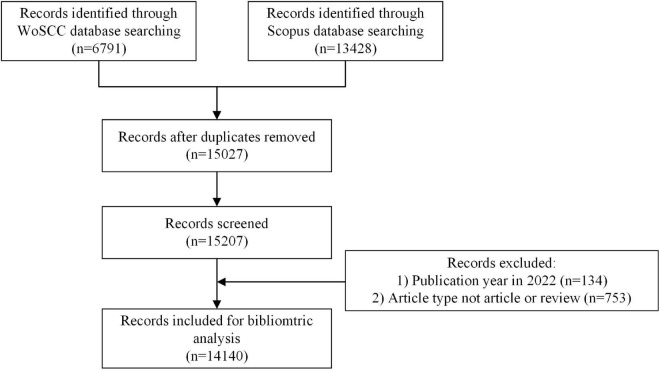
The flowchart of detailed screening steps of publications.

We collected the relevant literature from the Web of Science Core Collection (WoSCC) database^1^ and the Scopus database.^2^ Web of Science and Scopus are the two most massive and popularly used databases for bibliometric analysis. The following retrieval strategies were employed for the WoSCC database: TS = (connectom*) AND PY = (2005-2021). The following retrieval strategies were employed for the Scopus database: TITLE-ABS-KEY (connectom*) AND PUBYEAR > 2004 AND PUBYEAR < 2022 AND (LIMIT-TO (DOCTYPE, “ar”) OR LIMIT-TO (DOCTYPE, “re”)). Data from the WoS database included “title, abstract, author keywords, and Keywords Plus,” while data from the Scopus database included “title, abstract, and keywords.” There were no language limitations. All the processes of research and statistics were conducted on October 24, 2022, for eliminating the potential errors caused by daily updates of the database. Six thousand seven hundred and ninety-one records were identified through the WoSCC database and 13,428 records through the Scopus database. Duplicated records between the two databases were identified and excluded in R. The data retrieved were transformed to “bib” format and then imported into Rstudio 1.3.1. Records were excluded if publication year was in 2022 or article type was not article or review. Figure 1 showed the screening flowchart.

### 2.2 Quantitative analysis

The annual publication output on connectomes was illustrated. The most productive and most impactful journals were compared and ranked with their major subspecialties. Similarly, authors engaged in connectome research were ranked by their productivity and impact. The total number of publications was used to evaluate the productivity of journals, while the impact was assessed by h-index. The most productive and influential countries/regions were compared according to their total productions and average article citations. The country to which an article belongs was defined according to the country of the corresponding author. The countries/regions covered in the analysis were classified as developed or developing according to the *World Economic Situation and Prospects 2020* by United Nations (2020). The top 10 most productive institutions in connectome research were listed as well. Additionally, the collaborative relationship among countries/regions or different institutions was presented as a collaboration network map with lines that illustrated the frequency of cooperation between each entity. Finally, the top 20 most cited articles were summarized and analyzed.

### 2.3 Research topics and trends

The R package “bibliometrix” was used to visualize the research trends over the last decade (Aria and Cuccurullo, 2017). The main topics of the connectome were classified using Latent Dirichlet allocation (LDA) analysis. LDA is a three-layer Bayesian probabilistic model, which considers a document as a collection of unordered words and then identifies each topic as a probability distribution across words based on the co-occurrences of words (Schwarz, 2018). The R package “lda” was utilized to conduct an LDA analysis of the publications included. Words in keyword plus (author keywords, titles, and abstracts) were imported into LDA analysis. The number of topics was set to at least three. The raw data and code are available in the Supplementary Data Sheet 2.

## 3 Results

### 3.1 General information

**FIGURE 2:**
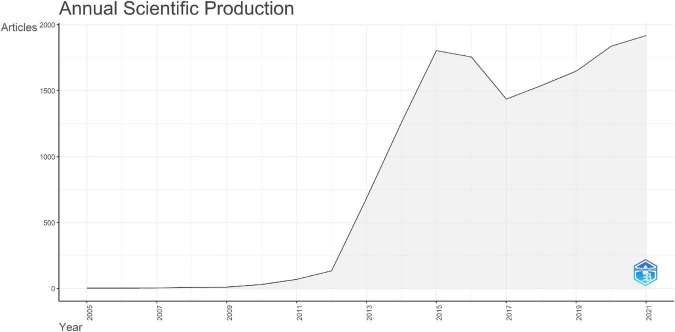
Annual publication production over time.

**FIGURE 3:**
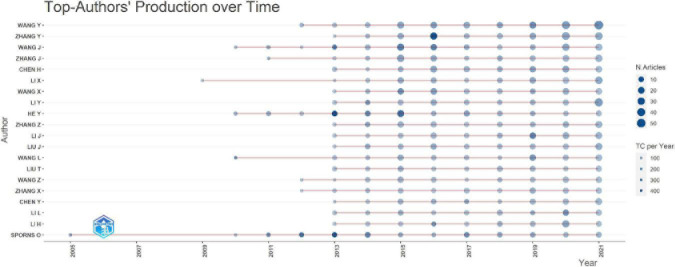
Top 20 productive authors in connectome research over time. N.Articles, number of articles; TC per Year, total citations per year.

A total of 14,140 publications on connectomes were included. The average number of citations per document was 36.91, a total of 759 sources were included. The annual production in the connectome field maintained a high growth trend since 2011 (Figure 2) with the annual growth rate reaching 53.59%. By 2021, the annual number of articles on connectomes reached 1,918. Detailed data for general information and annual production are available (see Supplementary Tables 1, 2). The production and citations trend of the top 20 most productive authors were shown in Figure 3. The size of the blue bubble indicated the number of articles, with the darker color indicating more total citations per year. Sporns O published his research in the field of connectomes as early as 2005. Since 2013, a significant number of researchers have invested in the research of connectomes and continue to work in this field.

### 3.2 Analysis of journals

Table 1 listed the top 10 productive and impactful journals in connectome research. The top 10 most productive journals contributed 42.86% (6060/14140) of the total publications. NEUROIMAGE ranked first with 1,427 articles, which was considerably ahead of the second-ranked journal (HUMAN BRAIN MAPPING, 714 articles). NEUROIMAGE also ranked first in terms of journal impact, with an h-index of 122. The growth trend of top journals was shown (see Supplementary Figure 1 and Supplementary Table 3). Six of the top 10 most productive journals and seven of the top 10 most influential journals in this field were in the neuroscience subspecialty. Comprehensive features, such as (a) the journal’s scope, (b) the average turnaround time, (c) the journal’s longevity, (d) whether it’s open access, and (f) publication fee, of the most productive journals were compared (see Supplementary Table 4).

**TABLE 1:** Top 10 productive and top 10 impactful journals in the field of connectomes.

Ranking	Journals ranked by articles	Neuroscience-subspecialty	Articles	Journals ranked by h-index	h-index	Neuroscience-subspecialty	TC*
1	NEUROIMAGE	Yes	1,427	NEUROIMAGE	122	Yes	76,894
2	HUM BRAIN MAPP	Yes	714	NEURON	91	Yes	26,932
3	PLOS ONE	No	439	J NEUROSCI	72	Yes	20,774
4	J NEUROSCI	Yes	402	PROC NATL ACAD SCI U S A	71	No	20,609
5	SCI REP	No	337	HUM BRAIN MAPP	63	Yes	17,847
6	NEUROIMAGE-CLIN	Yes	334	CEREB CORTEX	58	Yes	12,876
7	CEREB CORTEX	Yes	268	PLOS ONE	58	No	14,819
8	NEURON	Yes	220	NAT NEUROSCI	56	Yes	13,591
9	ELIFE	No	216	BRAIN	52	Yes	8,874
10	PROC NATL ACAD SCI U S A	No	203	BIOL PSYCHIATRY	46	No	6,642

*TC, total citations.

### 3.3 Analysis of countries and institutions

The 20 most productive and impactful countries/regions that participated in connectome research were listed in Table 2. Articles of the top 20 countries/regions (12,499) were equal to 88.39% of all 14,140 publications. The United States was the leading producer (4,868 articles) of scientific publications in this field, followed by China (1,681 articles) and Germany (945 articles). The developed countries published 73.84% (10,441/12,499) of articles among all the top 20 countries/regions. The top impactful countries/regions were ranked by average article citations (Ding et al., 2021). Pakistan ranked first in terms of average article citations.

**TABLE 2:** The most productive and impactful countries/regions in the field of connectomes.

Ranking	Countries/Regions ranked by articles	Articles	Developed	Countries/Regions ranked by average article citations	TC*	AAC*	Developed
1	USA	4,868	Yes	PAKISTAN	131	131	No
2	CHINA	1,681	No	LUXEMBOURG	213	71	Yes
3	GERMANY	945	Yes	UNITED KINGDOM	48,838	55.43	Yes
4	UNITED KINGDOM	881	Yes	VENEZUELA	51	51	No
5	CANADA	600	Yes	SWEDEN	4,757	45.74	Yes
6	ITALY	516	Yes	USA	222,556	45.72	Yes
7	AUSTRALIA	427	Yes	GEORGIA	592	45.54	No
8	NETHERLANDS	399	Yes	SAUDI ARABIA	313	44.71	No
9	FRANCE	395	Yes	NETHERLANDS	17,516	43.9	Yes
10	JAPAN	321	Yes	SWITZERLAND	12,002	43.49	Yes
11	SPAIN	286	Yes	FRANCE	15,734	39.83	Yes
12	SWITZERLAND	276	Yes	BELGIUM	4,893	38.83	Yes
13	KOREA	264	Yes	AUSTRALIA	14,841	34.76	Yes
14	BELGIUM	126	Yes	FINLAND	1,863	34.5	Yes
15	ISRAEL	118	No	GERMANY	32,543	34.44	Yes
16	SWEDEN	104	No	CANADA	20,154	33.59	Yes
17	INDIA	79	No	SINGAPORE	2,343	33	Yes
18	BRAZIL	76	No	ISRAEL	3,832	32.47	No
19	SINGAPORE	71	Yes	NEW ZEALAND	738	32.09	Yes
20	HUNGARY	66	Yes	AUSTRIA	2,038	31.35	Yes

*TC, total citations; AAC, average article citations.

**FIGURE 4:**
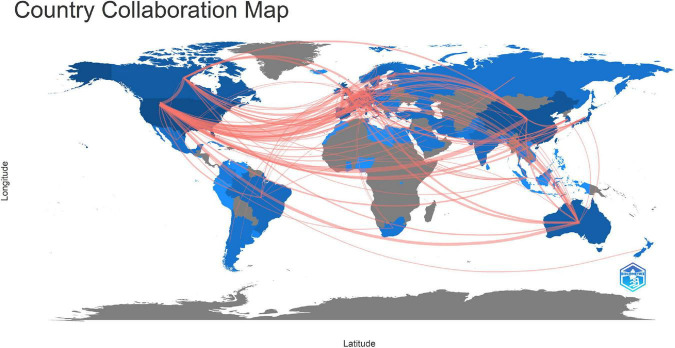
Collaboration network of countries/regions participating in connectome research.

The international collaboration in the connectome field was shown in Figure 4. The countries/regions that published articles on connectomes were marked in blue, with darker colors indicating more publications. Countries/regions that had cooperation were linked by red lines, while the width of the red line was proportionate to the number of collaborations. The United States had the most scientific research cooperation links with other countries, with the most frequent collaboration with European countries. Additionally, Canada, Australia, China, South Korea, Brazil, and Japan were among the prominent countries that have intensive international collaboration.

**FIGURE 5:**
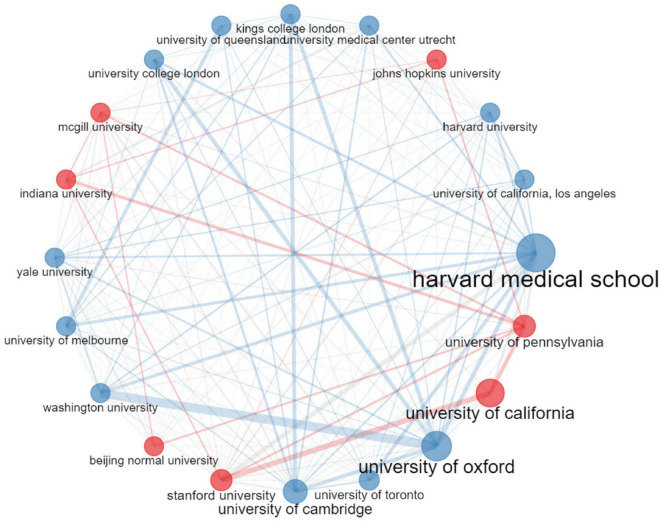
Collaboration network of institutions engaged in connectome research.

Table 3 listed the top 10 most productive institutions in connectome research around the world. The Harvard Medical School was ranked first with a total of 890 articles. The University of Pennsylvania came in second with 795 articles. Half of the top 10 most productive institutions were in the United States, while others came from the United Kingdom, China, Canada, and Australia. There was an extensive intertwined network of collaboration among the various institutions involved (Figure 5). As shown in Figure 5, institutions that cooperated frequently were clustered together to form two different colored set clusters. Affiliations with cooperation were connected by lines. The size of the bubble denoted the number of publications while the width of the lines reflected the frequency of scientific collaboration. As can be seen, several institutions have established significant partnerships in connectome research.

**TABLE 3:** Top 10 productive institutions in connectome research.

Ranking	Institutions	Articles	Country
1	Harvard Medical School	890	The United States
2	University of Pennsylvania	795	The United States
3	University of California	739	The United States
4	University of Oxford	605	The United Kingdom
5	University of Toronto	588	Canada
6	University of Cambridge	541	The United Kingdom
7	Stanford University	524	The United States
8	Beijing Normal University	516	China
9	Washington University	374	The United States
10	University of Melbourne	358	Australia

### 3.4 Topics and trends

**FIGURE 6:**
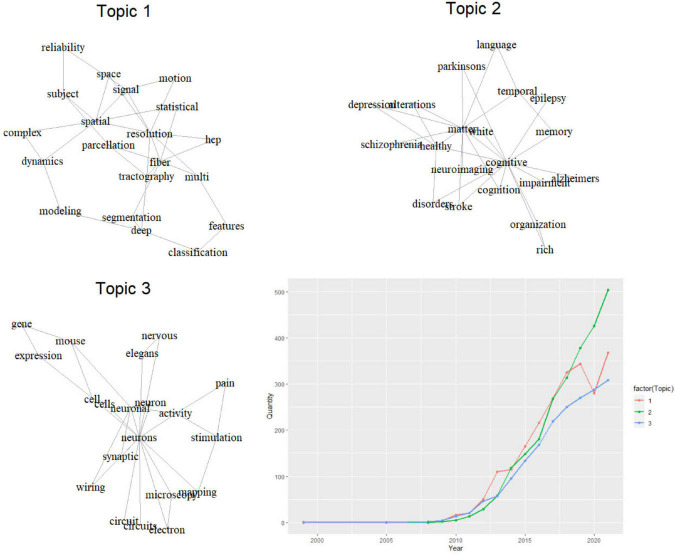
Research topics of connectomes over time. hcp, human connectome project.

Articles without abstracts were excluded and a total of 6,607 publications were included in the LDA analysis of keyword plus (author keywords, titles, abstracts). Three primary topics of research in this field were identified and designated as “Topic 1: Neuroimaging Analysis Technology,” “Topic 2: Function and Disease,” and “Topic 3: Basic research” (Figure 6). These three topics accounted for 35.0, 37.0, and 28.0% of the articles, respectively. Furthermore, we illustrated the annual volume of publications on these three topics. Between 2011 and 2018, the number of articles on the three topics increased significantly. Among them, the number of research devoted to neuroimaging analysis technology (Topic 1) had always been ahead, while the number of articles on related basic research (Topic 3) concerning cells, neural circuits, genes, etc. was relatively smaller. After 2019, the incremental pace of growth of Topic 1 and Topic 3 slightly slowed down, meanwhile, Topic 2 began to outrun the previous two topics, with an increasing amount of research examining alterations in brain connectivity in relation to cognitive, memory, and related neurological or psychiatric diseases. By 2021, the number of studies on Topic 2 had far surpassed that of associated analysis technology and basic research.

**FIGURE 7:**
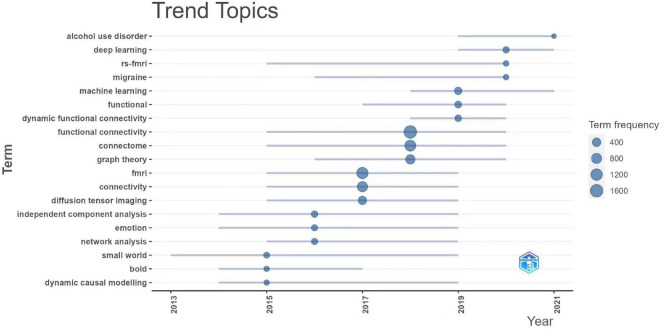
Recent research trend topics of connectomes. fmri, functional magnetic resonance imaging; rs-fmri, resting-state functional MRI; bold, blood oxygenation level dependent.

We assessed the connectome research trends using term frequency over the last 7 years (Figure 7). Among the high-frequency terms, computational or mathematical algorithms, including machine learning, deep learning, and graph theory had become the hotspots of connectome research. Alcohol use disorder and migraine have garnered an increasing amount of interest from researchers in the past 2 years. Resting-state fMRI and dynamic functional connectivity remain to be the preferred study approach and perspective in recent years.

### 3.5 Most cited articles

The analysis of local citations of articles enabled us to identify the most fundamental or significant studies in a specific research field. The top 20 most cited articles in connectome research were listed in Table 4. The most cited article was published by Van Essen et al. (2013) with 941 citations. NEUROIMAGE contributed seven of the top 20 most cited articles. The majority (16/20) of references were classified as Topic 1 (Neuroimaging Analysis Technology). In addition, we ranked the highly cited articles by global citations, and the results were presented in the Supplementary Table 5.

**TABLE 4:** Top 20 most cited articles in connectome research.

Ranking	Article_doi	Corresponding author	Local citations	Journal	Published year	Topic*
1	10.1016/j.neuroimage.2013.05.041	Van Essen DC	941	NEUROIMAGE	2013	1
2	10.1016/j.neuroimage.2013.04.127	Glasser MF	833	NEUROIMAGE	2013	1
3	10.1371/journal.pcbi.0010042	Sporns O	739	PLOS COMPUT BIOL	2005	1
4	10.1152/jn.00338.2011	Yeo BTT	634	J NEUROPHYSIOL	2011	1
5	10.1523/JNEUROSCI.3539-11.2011	Van Den Heuvel MP	488	J NEUROSCI	2011	1
6	10.1016/j.neuroimage.2012.02.018	Van Essen DC	417	NEUROIMAGE	2012	1
7	10.1038/nature18933	Glasser MF	413	NATURE	2016	1
8	10.1038/nrn3214	Bullmore ET	412	NAT REV NEUROSCI	2012	1
9	10.1038/nn.4135	Finn ES	403	NAT NEUROSCI	2015	2
10	10.1371/journal.pone.0068910	Xia M	332	PLOS ONE	2013	1
11	10.1016/j.neuroimage.2013.05.039	Smith SM	328	NEUROIMAGE	2013	1
12	10.1073/pnas.0911855107	Biswal BB	307	PROC NATL ACAD SCI U S A	2010	1
13	10.1016/j.tics.2013.09.012	Van Den Heuvel MP	307	TRENDS COGN SCI	2013	1
14	10.1038/nature13186	Oh SW	303	NATURE	2014	3
15	10.1093/brain/awu132	Crossley NA	289	BRAIN	2014	1
16	10.1016/j.neuroimage.2013.05.033	Barch DM	283	NEUROIMAGE	2013	2
17	10.1038/nrn3901	Fornito A	277	NAT REV NEUROSCI	2015	2
18	10.1016/j.neuroimage.2013.11.046	Salimi-Khorshidi G	265	NEUROIMAGE	2014	1
19	10.1016/j.neuroimage.2013.05.079	Hutchison RM	257	NEUROIMAGE	2013	1
20	10.1089/brain.2011.0008	Friston KJ	233	BRAIN CONNECTIVITY	2011	1

*Topic of the document was classified according to the topics in Figure 6.

## 4 Discussion

To our knowledge, this is the first bibliometric study on connectomes. This study is expected to provide an overview and detailed analysis of the publications in the field of connectomes. It may serve as a valuable resource for researchers in gaining an understanding of the underlying concepts and fundamental researches in this field, as well as determining research directions, journal selection, and research collaboration.

The connectome has been a popular research subject over the last decade, with the total number of publications increasing rapidly yearly. Sporns et al. (2005) first introduced the concept of the connectome in 2005, but it wasn’t until 2011 that a large number of authors began devoting their time to connectome research. Both the quantity and the breadth of relevant articles have increased dramatically in the last decade.

We conducted a quantitative analysis of the productivity and impact of journals engaged in connectome research. NEUROIMAGE, HUAMAN BRAIN MAPPING, and JOURNAL of NEUROSCI were ranked in the top five both in terms of productivity and impact, and hence may be the preferred choice for researchers exploring the connectome’s research frontiers and submitting high-quality work. Additionally, as the top 20 journals were mostly focused on neuroscience, researchers could also follow other journals with a subspecialty in neuroscience, such as CEREBRAL CORTEX, NEURON, BRAIN, NATURE NEUROSCIENCE, and BRAIN CONNECTIVITY.

LDA (Latent Dirichlet Allocation) has been widely used in recent years for publication analysis (Shatte et al., 2019). It could be used to identify latent topic information in a large-scale document collection or corpus (Griffiths and Steyvers, 2004). This study discovered that the scientific output for all three topics increased dramatically from 2011. Until 2016, more research focused on techniques of neuroimaging analysis than on the other two topics. Numerous researchers have achieved important advancements and developments in brain image data processing and brain network connectivity analysis techniques (Yan et al., 2013b; Glasser et al., 2016). The mapping of the human brain parcellation atlas has contributed significantly to the advancement of connectomes (Glasser et al., 2016). Machine learning and deep learning algorithms were widely used to build classification or prediction models with brain connectivity data (Horn et al., 2017; Neher et al., 2017). Along with the exploration of technology, an increasing number of researchers started to concentrate on the relationship between brain connectivity and brain function or diseases. The most extensively studied brain functions were cognition (Mill et al., 2017; Malagurski et al., 2020), memory (Ousdal et al., 2020; Barron et al., 2021), and language (Dick et al., 2014; Kaestner et al., 2020). In terms of diseases, many researchers have investigated the association between brain connectivity and epilepsy (Bernhardt et al., 2019; Moguilner et al., 2021), and stroke (Silasi and Murphy, 2014; Koch et al., 2021). In addition, some researchers have found alterations in brain connectivity in neural degenerative disorders, such as Parkinson’s disease (Horn et al., 2017; Krismer and Seppi, 2021) and Alzheimer’s disease (Wang et al., 2020). Schizophrenia (Lynall et al., 2010; Dong et al., 2018) and depression (Yun and Kim, 2021) were the most extensively studied psychiatric disorders in the study of connectomes. The number of studies on the topic of the underlying mechanisms of brain connectivity was less than those on the other two topics, but it was expanding gradually. Through experiments with elegans (Towlson et al., 2013; Taylor et al., 2021) or mouses (Oh et al., 2014), researchers have been able to investigate the mechanisms of neuronal cell potential changes, synaptic activity, and neuron circuits associated with brain connectivity at the cellular level. Similarly, some studies sought to discover how genetic variables influence brain connection and whether they could serve as a biomarker for neural diseases (Thompson et al., 2013; Yu et al., 2021).

By summarizing the trend of topic terms over the last few years, we may better understand the evolution of research hotspots over time, which can hopefully guide future research of connectomes. According to the results, the most recent research trend in the field of connectomes over the last 5 years has been cross-disciplinary research combined with various computer-science approaches such as machine learning and deep learning. Since the year 2020, numerous researchers have started to pay more attention to the abnormalities in brain connectivity and neural network mechanisms in alcohol use disorders (Elton et al., 2021; Gerchen et al., 2021) and migraine (Burke et al., 2020; Coppola et al., 2020). It has also been shown that dynamic functional connectivity analysis has become a popular issue in brain connectivity research. Most previous studies on functional connectivity potentially assumed that signals from specific brain regions remain consistent across time in the same task condition. The dynamic functional analysis challenges this fundamental premise by investigating changes in functional signals over time, which is more consistent with the features of brain networks and introduces new insights to the study of brain connectomes (Hutchison et al., 2013). The cross-fertilization of brain connectomes with graph theory was of particular interest, which has become a hot topic. Graph theory, as a branch of mathematics, entails the construction of nodes and connected edges to investigate various elements and their interactions (Sporns, 2018). This theoretical system fits fairly well with the way the brain is connected (Fornito et al., 2013). Using graph theory methods, we may topologically model complex brain network systems and analyze their efficiency and tightness at multiple scales, including local, global and modular, to better characterize the patterns of structural and functional brain connections (delEtoile and Adeli, 2017). This has contributed significantly to our understanding of the structure, development, and evolution of brain networks.

Given that the evolution of connectomes is inextricably linked to the exploration and innovation of a vast number of research in the field of analysis technology (Topic 1) over the years, here we highlighted some milestone research in the fundamental concept and connectome analysis techniques. In 2005, Sporns et al. (2005) proposed the term “connectome” to refer to the human brain’s complete structural map. There were only a handful of articles (He et al., 2007; Lichtman and Sanes, 2008; Seung, 2009) concerning the connectome before 2009, owing to a number of significant obstacles in mapping the connectome, such as capturing network connections at different spatial scales, explaining individual variability and structural plasticity, as well as elucidating the connectome’s function in determining brain dynamics (Sporns, 2013). In 2010, scientists from 35 centers across the world collaborated to provide global access to the 1,000 Functional Connectomes Project dataset, which contains resting-state functional MRI data from 1,414 participants, opening a new chapter in the science of human brain function (Biswal et al., 2010). Since 2011, a variety of connectome-related analysis techniques were proposed by researchers. These studies set the experimental foundation for following mechanistic and clinical studies and being heavily cited by publications in the field of connectomes. Glasser et al. (2013) provided the minimal pipelines for the preprocessing of various MRI data which were developed by the Human Connectome Project. The pipelines were widely used as a preprocessing standard in subsequent brain imaging and brain connectome research. Van Dijk et al. (2012) investigated the influence of head motion on fMRI measurements, concluding that head motion could significantly and systematically affect the fMRI network measurements and should be fully considered when analyzing the results. Similarly, Yan et al. (2013a) comprehensively assessed the impact of head micromovements on the outcomes of functional connectivity studies, providing a great reference value for subsequent experimental design and result discussion. van den Heuvel and Sporns (2011) further proposed the concept of “rich club” on the basis of topological research on brain networks, which gave an essential perspective for the study of the structural brain network. Xia et al. (2013) developed BrainNet Viewer, a graph-theoretic network visualization toolbox, to depict human connectomes as simplified models with balls and sticks. This MATLAB-based program has become one of the most popular for visualizing brain networks. Andersson and Sotiropoulos (2016) created a technique for estimating and correcting subject motion and distortions in diffusion imaging. It has been extensively used in the majority of DTI-based network studies.

There may be some limitations to the current study. Firstly, we only analyzed publications from the WoSCC and Scopus databases, which means that publications not indexed in these two databases were excluded and citations may have been underestimated. Secondly, rather than assessing whole texts, the study mainly evaluated selected information from the included publications, such as abstracts, titles, and author’s keywords, etc. Moreover, we included data till 2021, although the databases were constantly updated, so the most recent trends in 2022 may not be reflected. Last but not least, we did not evaluate the impact of the COVID-19 pandemic on the publications of connectome research, though there was much evidence indicating that the breakout of the epidemic has had a significant influence on academic research (Aggarwal et al., 2021; Malekpour et al., 2021).

## 5 Conclusion

In conclusion, the study illustrated a comprehensive overview of the connectome research. The productivity and impact of various academic journals, countries/regions, and institutions were summarized, which could be a guide for journal selection and international collaboration for researchers interested in connectomes. Brain functions and neuropsychological diseases were the major topics, followed by neuroimaging analysis technology. The combination with computational analysis algorithms, such as machine learning or deep learning, has been the most recent trend in the study of the connectome. Similarly, recent research has concentrated on the topic of dynamic functional connectivity and graph theory. With the quantitative and comprehensive analysis of the publication on connectomes, this study could provide researchers with some insight into the overview and hotspots in this certain field and shed new light on future research strategies.

## Data availability statement

The original contributions presented in this study are included in this article/Supplementary material, further inquiries can be directed to the corresponding authors.

## Author contributions

YY: concept, data acquisition, data interpretation, drafting, and manuscript preparation. GF: design, data acquisition, data analysis, manuscript editing, and funding acquisition. XL: concept, data interpretation, definition of intellectual content, and manuscript review. XZ: manuscript review, funding acquisition, and supervision. All authors contributed to the article and approved the submitted version.

## Footnotes

1
https://www.webofscience.com/

2
https://www.scopus.com/
