# Supplementary material for: Research trends and hotspots on connectomes from 2005 to 2021: A bibliometric and latent Dirichlet allocation application study
Source: Front Neurosci. 2022 Dec 22;16:1046562. doi: 10.3389/fnins.2022.1046562 (PMC9814013; doi:10.3389/fnins.2022.1046562)
Supplement: Supplementary file 1 [file Data_Sheet_1.pdf]

Supplementary materials for "Research Trends and Hotspots on Connectomes from 2005 to 2021: A Bibliometric and Latent Dirichlet Allocation Application Study"

Suppl.table 1. The main information of publications in connectomes

**MAIN INFORMATION ABOUT DATA**	
Timespan	2005:2021
Sources (Journals, Books, etc)	1357
Documents	14140
Annual Growth Rate %	53.59
Document Average Age	4.67
Average citations per doc	36.91
References	684805
**DOCUMENT CONTENTS**	
Keywords Plus (ID)	32068
Author's Keywords (DE)	20238
AUTHORS	
Authors	43415
Authors of single-authored docs	548
**AUTHORS COLLABORATION**	
Single-authored docs	632
Co-Authors per Doc	6.26
International co-authorships %	15.69
**DOCUMENT TYPES**	
article	12231
review	1909

Suppl.table 2. Annual production of connectome research

Year	2005	2006	2007	2008	2009	2010	2011	2012	
Articles	2	2	4	9	10	30	69	134	
Year	2013	2014	2015	2016	2017	2018	2019	2020	2021
Articles	684	1259	1804	1756	1436	1538	1648	1837	1918

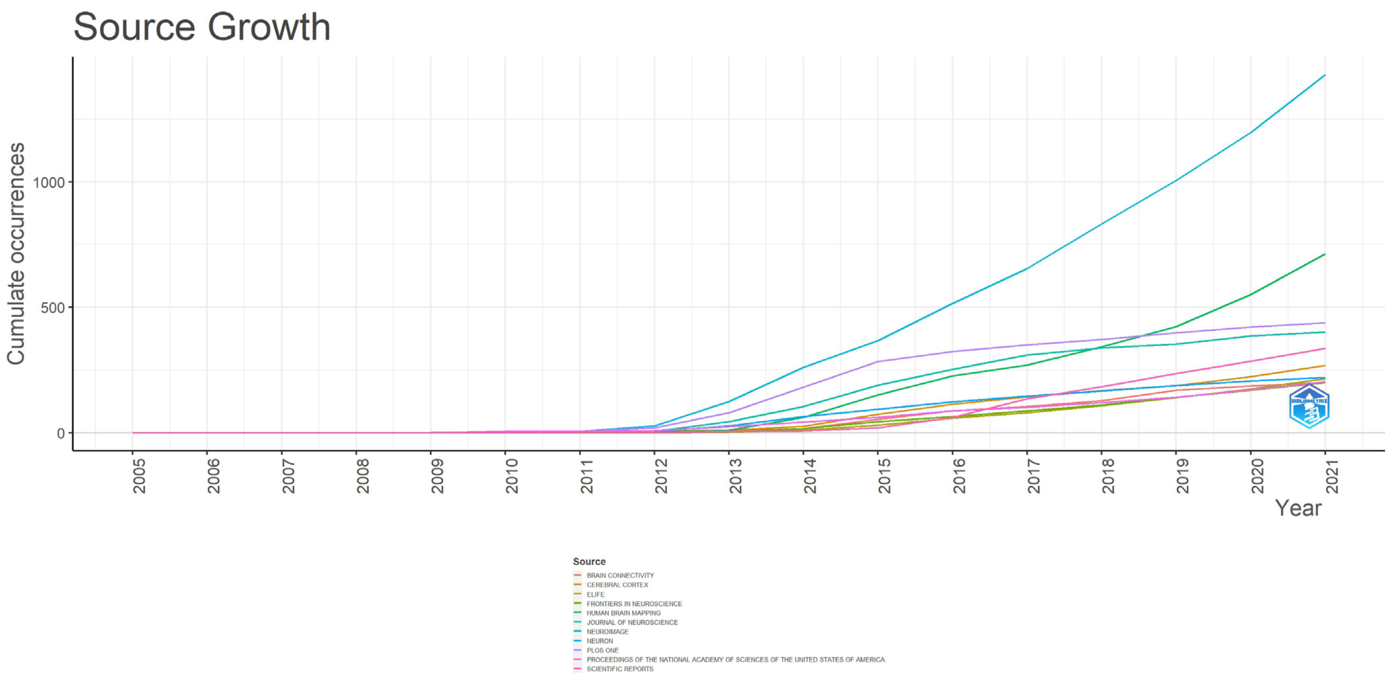

Suppl.figure 1. The annual growth trend of the top 10 productive journals

Suppl.table 3. Annual production of the top 10 productive journals from 2005 to 2021

Year	NEUROIMAGE	HUM BRAIN MAPP	PLOS ONE	J NEUROSCI	SCI REP	CEREB CORTEX	NEURON	ELIFE	PROC NATL ACAD SCI USA	BRAIN CONNECT
2005	0	0	0	0	0	0	0	0	0	0
2006	0	0	0	0	0	0	0	0	0	0
2007	0	0	0	0	0	0	0	0	0	0
2008	0	0	0	0	0	0	0	0	0	0
2009	0	0	1	0	0	0	1	0	0	0
2010	0	1	1	1	0	0	2	0	5	0
2011	6	1	5	4	1	2	2	0	5	3
2012	27	2	19	5	1	7	6	0	9	4
2013	125	11	80	45	5	11	27	2	25	7
2014	261	62	182	104	8	26	65	11	43	16
2015	367	151	284	190	19	73	94	31	62	53
2016	515	226	324	253	61	114	123	58	87	88
2017	655	270	350	310	136	143	146	80	101	105
2018	832	342	372	338	184	168	167	107	120	128
2019	1005	423	398	354	236	188	188	140	141	170
2020	1198	551	422	386	286	224	207	174	171	187
2021	1427	714	439	402	337	268	220	216	203	200

Suppl.table 4. Comprehensive features* of the top 10 productive journals.

Journal	Scope	average turnaround time(first decision)	Longevity	Open Access	Publication Fee
NEUROIMAGE	NEUROIMAGE is a Journal of Brain Function provides a vehicle for communicating important advances in acquiring, analyzing, and modelling neuroimaging data and in applying these techniques to the study of structure-function and brain-behavior relationships.	5.6 weeks	since 1992	yes	$3,450
HUM BRAIN MAPP	Human Brain Mapping publishes peer-reviewed basic, clinical, technical, and theoretical research in the interdisciplinary and rapidly expanding field of human brain mapping. The journal features research derived from non-invasive brain imaging modalities used to explore the spatial and temporal organization of the neural systems supporting human behavior.	-	since 1993	yes	$3,300
PLOS ONE	PLOS ONE welcomes original research submissions from the natural sciences, medical research, engineering, as well as the related social sciences and humanities.	43 days	since 2006	yes	$1,805
J NEUROSCI	JNeurosci is a multidisciplinary journal that publishes papers on a broad range of topics of general interest to those working on the nervous system.	32 days	since 1981	yes	$2,270
SCI REP	Scientific Reports is an open access journal publishing original research from across all areas of the natural sciences, psychology, medicine and engineering.	56 days	since 2011	yes	$2190
CEREB CORTEX	Cerebral Cortex publishes papers on the development, evolution, organization, plasticity, and function of the cerebral cortex, including the hippocampus. Studies with clear relevance to the cerebral cortex, such as the thalamocortical relationship or cortico-subcortical interactions, are also included.	-	since 1991	yes	$3450
NEURON	Neuron has established itself as one of the most influential and relied upon journals in the field of neuroscience.	4–5 weeks	since 1988	yes	$8,900

	The editors embrace interdisciplinary strategies that integrate biophysical, cellular, developmental, and molecular approaches with a systems approach to sensory, motor, and higher-order cognitive functions. Neuron serves as one of the premier intellectual forums of the entire neuroscience community.				
ELIFE	eLife is a selective journal that publishes promising research in all areas of biology and medicine.	43 days	since 2003	yes	$3,000
PROC NATL ACAD SCI USA	PNAS publishes exceptional research in all branches of the Biological, Physical, and Social Sciences. Innovation often happens at the margins, and we are particularly interested in research that crosses disciplinary bounds, answers questions with broad scientific impact, or breaks new ground.	46 days	since 1915	yes	$2,595
BRAIN CONNECT	Brain Connectivity is an international neuroscience journal dedicated to the publication of original research, communications, and scientific reviews concerned with all aspects of anatomical, functional, and causal connections between distinct units within the central nervous system. The journal will consider studies in both human and animal models.	-	since 2011	yes	$1,250

* all information obtained from the official website of each journal.

Suppl.table 5. Top 20 most cited articles (by global citations) in connectome research.

Ranking	Article_doi	Corresponding Author	Global citations	Journal	Published year
1	10.1152/jn.00338.2011	Yeo BTT	3754	NEUROIMAGE	2011
2	10.1016/j.neuroimage.2013.05.041	Van Essen DC	2347	NEUROIMAGE	2013
3	10.1073/pnas.1602413113	Eklund A	2141	PLOS COMPUT BIOL	2016
4	10.1016/j.neuroimage.2013.04.127	Glasser MF	2094	J NEUROPHYSIOL	2013
5	10.1073/pnas.0911855107	Biswal BB	1963	J NEUROSCI	2010
6	10.1371/journal.pone.0068910	Xia M	1918	NEUROIMAGE	2013
7	10.1089/brain.2011.0008	Friston KJ	1911	NATURE	2011
8	10.1371/journal.pcbi.0010042	Sporns O	1893	NAT REV NEUROSCI	2005

9	10.1038/nature18933	Glasser MF	1786	NAT NEUROSCI	2016
10	10.1038/nrn3214	Bullmore ET	1767	PLOS ONE	2012
11	10.1016/j.neuroimage.2011.07.044	Van Dijk Kra	1710	NEUROIMAGE	2012
12	10.1016/j.neuroimage.2013.05.079	Hutchison RM	1593	PROC NATL ACAD SCI U S A	2013
13	10.1523/JNEUROSCI.3539-11.2011	Van Den Heuvel MP	1414	TRENDS COGN SCI	2011
14	10.1016/j.clinph.2015.02.001	Rossini PM	1401	NATURE	2015
15	10.1016/j.neuroimage.2015.10.019	Andersson JLR	1374	BRAIN	2016
16	10.1152/jn.00783.2009	Van Dijk Kra	1298	NEUROIMAGE	2010
17	10.1038/nature13186	Oh SW	1257	NAT REV NEUROSCI	2014
18	10.1038/nature12107	Chung K	1250	NEUROIMAGE	2013
19	10.1093/brain/awt162	Leech R	1249	NEUROIMAGE	2014
20	10.1038/nn.4135	Finn ES	1196	BRAIN CONNECTIVITY	2015
